# The fourth delay and community-driven solutions to reduce maternal mortality in rural Haiti: a community-based action research study

**DOI:** 10.1186/s12884-018-1881-3

**Published:** 2018-06-20

**Authors:** Tonya MacDonald, Suzanne Jackson, Marie-Carmèle Charles, Marius Periel, Marie-Véna Jean-Baptiste, Alex Salomon, Éveillard Premilus

**Affiliations:** 1 School of Midwifery, Health Sciences Building, Laurentian University, 935 Ramsey Lake Road, Sudbury, ON P3E 2C6 Canada; 2 Dalla Lana School of Public Health, Health Sciences Building, University of Toronto, 155 College Street, Suite 526, Toronto, ON M5T 3M7 Canada; 3 Cégep du Vieux Montréal, Montreal, Canada; 4 Hôpital Albert Schweitzer, Deschapelles, Haiti

**Keywords:** CBAR, Maternal mortality, Fourth delay, Near-miss maternal experiences

## Abstract

**Background:**

In Haiti, the number of women dying in pregnancy, during childbirth and the weeks after giving birth remains unacceptably high. The objective of this research was to explore determinants of maternal mortality in rural Haiti through Community-Based Action Research (CBAR), guided by the delays that lead to maternal death. This paper focuses on socioecological determinants of maternal mortality from the perspectives of women of near-miss maternal experiences and community members, and their solutions to reduce maternal mortality in their community.

**Methods:**

The study draws on five semi-structured Individual Interviews with women survivors of near-misses, and on four Focus Group Discussions with Community Leaders and with Traditional Birth Attendants. Data collection took place in July 2013. A Community Research Team within a resource-limited rural community in Haiti undertook the research. The methods and analysis process were guided by participatory research and CBAR.

**Results:**

Participants identified three delays that lead to maternal death but also described a fourth delay with respect to community responsibility for maternal mortality. They included women being carried from the community to a healthcare facility as a special example of the fourth delay. Women survivors of near-miss maternal experiences and community leaders suggested solutions to reduce maternal death that centered on prevention and community infrastructure. Most of the strategies for action were related to the fourth delay and include: community mobilization by way of the formation of Neighbourhood Maternal Health/Well-being Committees, and community support through the provision/sharing of food for undernourished women, offering monetary support and establishment of a communication relay/transport system in times of crisis.

**Conclusions:**

Finding sustainable ways to reduce maternal mortality requires a community-based/centred and community-driven comprehensive approach to maternal health/well-being. This includes engagement of community members that is dependent upon community knowledge, political will, mobilization, accountability and empowerment. An engaged/empowered community is one that is well placed to find ways that work in their community to reduce the fourth delay and in turn, maternal death. Potentially, community ownership of challenges and solutions can lead to more sustainable improvements in maternal health/well-being in Haiti.

## Background

The United Nation’s fifth Millennium Development Goal aimed to reduce global maternal mortality by three-quarters between 1990 and 2015 [1]. Haiti is a low-income country where approximately 37% of births are attended by Skilled Health Personnel [2], 64% of births take place at home [3], less than 10% occur in a health facility for women of the lowest wealth quintile [4], and where the maternal mortality ratio is unacceptably high. According to 2015 WHO data, Haiti’s maternal mortality ratio indicated 359 [range 236–601] maternal deaths per 100,000 live births [4] although these data lack complete registration of maternal deaths. Recent literature shows Haiti’s poor progress towards reducing inequalities in reproductive health compared to other Latin American and Caribbean countries, and points to the need for renewed actions to bridge gaps in maternal health [5].

Determinants of maternal mortality have been described and discussed extensively in the literature. In 1991 Thaddeus and Maine introduced the Three Delays framework to describe obstetric obstacles that lead to maternal death [6]. Maternal death can result from the delay: (1) to seek appropriate medical help for an obstetric emergency; (2) to reach an appropriate obstetric facility; and (3) to receive adequate care at the facility [6–9].

In a comprehensive review of literature from 1980 to 2011, Pacagnella and group critiqued the Three Delays model for its retrospective approach, neglect to consider underlying factors that contribute to maternal death and lack of consideration for preventative care that reduces maternal mortality [9]. They and others have suggested that there might be a fourth delay related to community factors [9, 10].

In terms of literature specific to delays and maternal health in Haiti, Barnes-Josiah et al. applied Thaddeus and Maine’s Three Delays framework to understand the social and medical circumstances surrounding Haitian maternal mortalities [7]. They found that while most maternal deaths occur due to the first delay, that the third delay (to receive adequate care at an obstetric facility) was the most significant [7]. Barnes-Josiah and colleagues concluded that improving the maternity care system would most greatly impact maternal mortality in Haiti [7]. Furthermore, White et al.’s examination of health-seeking behaviours of pregnant women in rural Haiti found the first delay to be most significant in this community [11]. These authors emphasized that most interviewed participants lacked a certain awareness of their healthcare needs and relied on their partner’s/husband’s and/or mother’s advice regarding seeking care [11]. This highlights the need for culturally competent health promotion campaigns targeted at women and other key players in their social networks [11]. This study also considered how the socioecological context contributed to the delay to reach an appropriate obstetric facility [11]. Several researchers have investigated the determinants of institutional delivery in Haiti. Babalola, for example, investigated antenatal services and Skilled Birth Attendants to conclude that maternal health service use in Haiti must especially consider the needs of multiparous women, women with little or no education and must take into account accessibility of services for the poor and the “distance-decay” phenomenon in reaching services [12]. Babalola brings to light the importance of considering community mobilization efforts; these efforts allow a community to self-identify service-use norms and expectations within the community’s context [12]. No other literature about maternal health in Haiti was found where specific reference was made regarding this second delay of the Three Delays framework.

Literature on personal accounts of “near-miss”, community (user) perspectives, and community engagement to reduce maternal mortality in Haiti is limited. In terms of near-misses, women survivors of maternal experiences are a proxy group that often shares common characteristics with those who died “on the road of death” [9]. While these survivors have a unique perspective on maternal mortality that may contribute to insight on reducing maternal death [9], no studies were located that considered personal accounts of near-misses. Conversely, a few studies examined community views of Haitian women and healthcare workers as they relate to contraceptive needs in Northern Haiti [13], and a maternal/child health needs assessment [14] to help inform program development in maternal/child health. One study, by Small and colleagues in rural Haiti, highlighted the importance of community members’ engagement in maternal mortality reduction activities [15]. Small and team concluded that maternal mortality would be best addressed through systematic evaluation of hospital-based practices in collaboration with community-based strategies and driven by engaged community members [15].

**Fig. 1:**
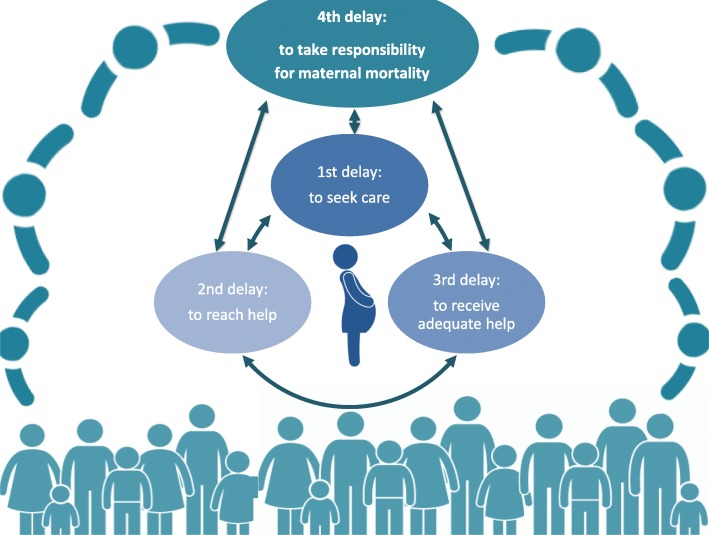
Maternal Mortality in a Community in Rural Haiti

A conceptual framework, Maternal Mortality in a Community in Rural Haiti (Fig. 1) was developed for our study as a visual representation of causal linkages together with their effects and impacts [16]. Our framework is underpinned by Social Constructivism and overlaps with theories of empowerment, participatory communication, social change, socioecology and public health. The framework represents a proximal focus- of causal linkages that correspond to community perspectives on why women die in pregnancy, during birth and the 42 days after giving birth in this rural Haitian community, and what can be done to reduce maternal mortality in the community; it links individual and micro-level factors that play a role and are influenced by modifying and confounding environmental and social factors that work at multiple levels [16].

In our conceptual framework, the large outer circle represents a community in rural Haiti, dispersed geographically, and impoverished by way of availability and accessibility to water, food, electricity, employment, healthcare, and transportation (to name a few). This community is made up of women, men, and many youth and children. The woman (and her maternal health) is at the heart of this rural Haitian community and of the framework. The issue of maternal mortality is greatly influenced by four delays as seen by the incorporation of Thaddeus and Maine’s Three Delays Framework, and the (recently added) Fourth Delay. Taken alone, either of the delays can lead to maternal death. Taken in combination or altogether, the four delays also contribute to maternal death. The representation illustrates a direct relationship between these delays and the community. The four delays are embedded within the community, influenced by the community and in turn impact the community as a whole.

The first to fourth delays are influenced by the socioecological determinants of a woman’s health, such as status of woman or socioeconomic factors like knowledge, age, economic status, and social support. The community’s resources and its engagement likewise impact the delays. Community resources refer to the availability of human and natural resources within the community and the infrastructure to access them. Community “resourcefulness” is affected by the distribution of resources which inevitably also plays a role in the socioecological determinants of maternal health. Community engagement here refers to the concept of community members’ collective commitment and involvement regarding the issue of maternal mortality and its reduction.

Finally, our conceptual framework aims to provide an hypothesis of the causal linkages of maternal mortality and their effects and impacts at the individual, family and community levels. In this framework, the fourth delay figures prominently and appears to exert a larger effect on maternal death in this rural Haitian community. The fourth delay represents the delay in which the most potential rests in the community’s hands because this delay has the potential to also impact (positively or negatively) the other three delays. Power derived at the community level stands the greatest chance for practical, achievable, immediate and sustainable changes. Positive change is influenced by community members’ knowledge of the issue of maternal mortality in their community, political will to bring about change, community efforts to mobilize; it is affected by the degree of accountability toward maternal death, and the community’s level of empowerment and belief that they can reduce maternal mortality.

Finally, the objective of this research was to explore maternal mortality in rural Haiti through Community-Based Action Research (CBAR). This paper focuses on the determinants of maternal mortality from the perspectives of women of near-miss maternal experiences and community members, and their solutions to reduce maternal mortality in their community. We hoped that sustainable ways to reduce maternal mortality could be found through a community-based/centred and community-driven comprehensive approach to maternal health/well-being.

## Methods

This study took place over three months in 2013, in a resource-limited rural community populated by 22,000 Haitians, many living in mountainous/hard-to-reach villages that line the Artibonite Valley of central Haiti. The community had one clinical dispensary that provided vaccination services, malnutrition screening, maternal healthcare and public health education. The dispensary was run by Hôpital Albert Schweitzer, a nongovernmental hospital serving a population of over 350,000 [11, 15, 17].

This CBAR study directly engaged members of the community to explore perspectives of their own community about maternal mortality. Our study design incorporated an Idealist paradigm in which the world is seen as socially constructed, built upon the thought that individuals interpret their own circumstances and a researcher (or research team) becomes part of the environment. The choice of a CBAR approach was justified by Small and colleagues’ study in rural Haiti [15] that highlighted the importance of community members’ engagement in maternal mortality reduction activities.

The initial step in our study was the formation of a community research team (the CRT). The CRT consisted of six members, literate in Haitian Creole and/or French, and included two Co-Leads (first author and Haitian (Htn) nurse-midwife) and four community members (health agent coordinator, pastor, homemaker and farmer). A skills training workshop facilitated by the Co-Leads aided the team’s acquisition of qualitative research skills and a deeper understanding of maternal mortality and the obstetric delays framework.

A total of 36 participants took part in either focus group discussions (FGD) or individual interviews (II). We convened 31 participants (adult women and men) in four focus groups with half from each of two different geographical parts of the community: two focus groups with Community Leaders (CL) (15 participants), and two focus groups with Traditional Birth Attendants (TBAs) known as ‘matwon’ (16 participants) (Table 1). The CRT undertook purposive sampling and direct/face-to-face recruitment to recruit 11 women and 4 men Community Leaders, and 12 women and 4 men TBAs. Focus group participants were from a broad range of age (25–65 years), community status and occupation.

**Table 1:** Features and characteristics of focus group discussions

Focus group discussion	Participant features	Participant ratio of women to men	Age range (in years)	Facilitator characteristics
FGD 1 Community Leaders (*n* = 7)	Teacher, volunteer health worker, farmer, pastor, homemakers	4:3	25–50	CRT Htn Co-Lead
FGD 2 Community Leaders (*n* = 8)	Merchant, volunteer health worker, elder, farmer, homemakers	7:1	25–40	CRT Member
FGD 3 Traditional Birth Attendants (*n* = 8)	All with some community training	5:3	35–65	CRT Htn Co-Lead
FGD 4 Traditional Birth Attendants (*n* = 8)	All with some community training	7:1	40–50	CRT Member

We also conducted five semi-structured individual interviews with adult women (W) of near-miss maternal experiences (Table 2) to gather their views about the causes of maternal mortality.

**Table 2:** Features and characteristics of individual interviews

Individual interview number	Participant characteristics	Age range (in years)	Interview location features	Interviewer characteristics
II 1	Mother: 1 infant (died at 3 months old) ● not partnered ● unemployed ● literate	20–25	Participant’s home, outdoors	CRT Htn Co-Lead
II 2	Mother: 3 living children ● partnered ● market merchant ● illiterate	25–35	Community church, outdoors; several hours walk from participant’s home	CRT Member
II 3	Mother: 3 living children + 1 miscarriage ● partnered ● homemaker ● literate	30–35	Participant’s home, outdoors	CRT Member
II 4	Mother: 2 living children + 1 stillborn ● partnered ● homemaker ● illiterate	25–35	Participant’s home, outdoors	CRT Member
II 5	Mother: 1 stillborn + 1 neonatal death ● partnered? ● market merchant ● illiterate	25–35	Community church, outdoors; within participant’s neighbourhood	CRT Member

A list of potential interview participants was generated with the help of dispensary staff and CRT from women survivors in the catchment area who ranged in age (20–35 years) and different stages of near-miss experience (i.e. in pregnancy, during birth, and in the first 42 days postpartum). Women on this list were approached by a CRT member and invited to participate in interviews. Five women survivors were recruited.

The focus group discussions/interviews were led by different Haitian members of the CRT (with the first author always present as an active observer) and took place at various secure and convenient locations within the community. Individual interviews took place at or near the participant’s home. Participants were asked what they believed were causes of maternal mortality in their community, community impact of maternal death, and proposed solutions to reduce maternal mortality within their community. Similarly, interview participants were asked about their unique, near-miss maternal experiences, challenges encountered, support received, and lessons learned (see Table 3).

**Table 3:** Key questions covered in focus group discussions and individual interviews

Data Collection/Participants	Question Guides
FGD 1 + 2 Community Leaders (*N* = 15)	1. As CLs, what is your understanding of maternal mortality? 2. As CLs, what is your experience of why women in your community die in pregnancy, during birth and in the six weeks following giving birth? 3. From your knowledge and experience, what are the causes for women dying in pregnancy, during birth and in the 42 days after giving birth? 4. How do women in the community know when they are having problems in their pregnancy, during labour/birth or for the weeks following birth? 5. In our community, what does the community do to help women in pregnancy, during labour and birth and after birth? 6. As CLs, what help can you give/do you give for women having these kinds of problems? 7. What is the impact on our community when women die, either in pregnancy, while giving birth, or in the 42 days after having a baby? 8. As CLs, what can you propose for our community as ways to stop women dying at this time in their lives?
FDG 3 + 4 Traditional Birth Attendants (*N* = 16)	1. As TBAs, what is your understanding of maternal mortality? 2. In our community, what do you do to help women in pregnancy, during labour and birth and after birth? 3. When things are not going well in pregnancy, labour and birth or the weeks after having a baby, how does that look? 4. As TBAs, how do you know when women in the community are having problems in their pregnancy, during labour/birth or for the weeks following birth? 5. In our community, as TBAs what do you do to help women in pregnancy, during labour and birth and after birth, when they are having problems? 6. As TBAs, what is your experience of why women in your community die in pregnancy, during birth and in the six weeks following giving birth? 7. What is the impact on our community when women die, either in pregnancy, while giving birth, or in the 42 days after having a baby? 8. What help could the whole community give so women don’t die in our community? 9. As TBAs, what can you propose for our community as ways to stop women dying at this time in their lives?
IIs 1–5 Women Survivors of Near-Miss Maternal Experiences (*N* = 5)	1. What was it like for you to be pregnant, to be in labour/give birth, and/or during the days/weeks after you had your baby? 2. What challenges or difficulties did you experience in your pregnancy, during birth or in the weeks after having a baby? 3. How did you know when something wasn’t going well for you? 4. What did you do to try to “fix the problem”? 5. What help did you get from those around you? From your community? 6. How did people from your community help you? 7. How do you feel about this experience of pregnancy/birth/weeks after birth? 8. What did you learn from this difficult experience? 9. What advice can you give others (other women, other community members), given the difficult experience you have had?

Legend: *FGD* Focus Group Discussion, *CLs* Community Leaders, *TBAs* Traditional Birth Attendants, *IIs* Individual Interviews

Focus groups/interviews lasted between 25 min and 1 1/2 h. With participants’ permission, all discussions/interviews were digitally recorded and transcribed verbatim in Haitian Creole by a Creole-French-speaking Haitian with professional experience in transcription. The nine transcripts were independently reviewed by the Co-Leads to ensure accuracy of transcription.

A participatory qualitative analysis group process [18] was adapted and employed by the CRT. The group analysis process (in Haitian Creole) involved four phases:

In Phase 1 (Preanalysis Step), the Co-Leads colour-coded the transcripts by focus group number and cut print-outs of the participants’ sentences and/or paragraphs into strips and bundled them into categories corresponding to the three guiding questions: a) Why do women die in pregnancy, during birth and the 42 days after birth in the community? b) What is the impact of maternal death on the community? c) What solutions can be offered to reduce maternal mortality in the community? In Phase 2 (Grouping Data and Identifying Themes) the CRT grouped the strips in each category into themes and subthemes, in relationship to the guiding questions and the study’s research questions. In this phase, the CRT also identified key quotes that best illustrated the community’s views.

Interview data were similarly analyzed for Phases 1 and 2 using Jackson’s group process [17]. The CRT decided to analyze the interviews one at a time, and focused on three themes: a) woman’s near-miss maternal experience b) woman’s experience of community help and c) woman’s proposed solutions to reduce maternal mortality in her community. The CRT used the themes and subthemes that emerged from focus groups, and looked for similarities in the interviews. Outlying themes/subthemes were noted. Key quotes were identified for use in the final report for the voices of the individual interview participants to be heard and honoured.

Phases 3 and 4 of Jackson’s process [18] required making sense of the themes and making interconnections between and across all data. The Co-Leads completed these phases.

## Results

Results are presented under the themes that emerged from data analysis, and guided by the socioecological determinants of (maternal) health and the four delays conceptual framework. The first delay relates to the decision to seek help during an obstetrical emergency; the second- a delay to receive help; the third- a delay to receive adequate help; and fourth- a delay to take responsibility/to be accountable for maternal death.

### 1st delay: Decision to seek help

In our study participants were very aware that pregnancy hypertension/eclampsia, hemorrhage, anemia and obstructed labour contributed to maternal mortality in their community. According to a woman survivor, maternal mortality is caused by:

*Eclampsia kills babies and mothers… and when you are pregnant and you don’t feel well, go see the doctor; your feet are swollen, go to the doctor; you have a headache, go to the doctor; you have a problem “below”, go to the doctor; for all problems you have it’s going to the doctor’s that will resolve the problems.* –Mother of Two Children (one stillborn and one neonatal death) (W2)

Within focus group discussions, participants introduced the idea of “negligence” and its role in maternal outcomes. They described this concept as a woman’s lack of awareness of and/or denial of pregnancy danger signs/symptoms, waiting too long before seeking care for these, or negligence to seek healthcare in pregnancy at all. Participants felt that knowledge impacted maternal death because women (especially youth in the community) lacked knowledge/awareness (of maternal health/well-being, of community prenatal care and social services), were misinformed of pregnancy/birth/afterbirth or were “negligent” in seeking appropriate healthcare advice. These participants also linked youth to having a lesser degree of maternal health knowledge, increased fear/level of stress in pregnancy which included hiding a pregnancy from one’s partner/family/friends, lesser degree of partner involvement and reduced ability to make informed choices. Focus group participants identified several concepts that may be related to the first and fourth delays-delays in decisions to seek help and/or in the community’s accountability with respect to maternal death where delays have occurred.

Economic status of the woman or her family played a significant role and contributed to different delays in the decision to seek help. One woman related economic means and family planning:

*It’s that you should go to the hospital [to deliver]. Well sometimes you are home and you don’t have money to go to the hospital. Your husband doesn’t have money for you to go so if you don’t go, the baby can get really big in your belly and you don’t go to the hospital so you won’t know what kind of baby you are having, and if it will die….well I say that problems can happen if you have more children so it’s better to use family planning.* -Mother of Three Living Children (W1)

Focus group participants discussed socioecological status of the woman and linked these to women’s stress of an unwanted pregnancy (by youth and by adults already with children), the decision to conceal or terminate a pregnancy (due to financial worries), and family burden of an additional child. Women’s degree of independence was associated with decisions about abortion, seeking care and leaving home for hospital-based care. Women survivors of near-misses felt obliged to await the return of their partners (from the field, another community, the ‘boko’ (Haitian Voodoo sorcerer) or ‘houngan’ (Haitian Voodoo male priest) before a critical decision about their health/well-being could be made, leading to serious delays of the first type.

### 2nd delay: To reach help

Participants directly related maternal death from hemorrhage to the second delay. Many participants described hemorrhage (e.g. from retained products of conception or the use of various folk/spiritual rituals) contributed as a leading cause of mortality in the community. They cited causes of maternal death as distance travelled to reach help, and time taken before receiving adequate care to treat blood loss.

Of the five women survivors of near-misses, one participant had complications due to an incomplete miscarriage that occurred at home in mid-pregnancy. When asked if she could have died from her situation she responded:

*It is the road to death, yes when you lose a lot of blood and you hemorrhage; now you can die if you lose a lot of blood until you have none left and you die, yes.* -Mother of Three Living Children (W1)

For survivors of near-misses, hypertension and eclampsia figured prominently with respect to reaching help. Three of the participants suffered severe complications of hypertension during their pregnancies and were transported to the hospital, each suffering from eclamptic seizures en route. They gave birth at the hospital: one delivered a live infant; the second delivered a live infant by caesarean section but the infant died at three months of age for reasons unknown. The third woman was delivered by caesarean section and her infant died shortly after birth. This participant also reported suffering from hypertensive seizures in the early postpartum period, an incisional infection, and ongoing visual disturbances.

Focus group participants often discussed economic status with respect to age (i.e. being more precarious for pregnant unemployed youth), and related this to the second and third delays. In general, economic instability impacted the ability to have work in the first place, seek or pay for maternal healthcare, eat well/at all, or pay for transportation to a healthcare facility. This is exemplified in a female Community Leader’s words:

*People have economic problems, they don’t have money and they can die while somebody gets others to carry her, she can die, she can die because the road is too long to get to the dispensary, and then they don’t take care of her. She can die. Those are all the problems we see here.* (CL2)

### 3rd delay: To receive adequate help

In our study, participants from each focus group made direct ties between community infrastructure and healthcare services at the dispensary or hospital as they related to receiving adequate help. They stated that healthcare services were inadequate, incomplete and inappropriate in their community citing a lack of adequately skilled maternal care providers, which included TBAs and dispensary staff alike. Nonetheless, participants from three of the focus groups regarded the provision of maternal healthcare as a form of community help to women, and encouraged community members to seek healthcare services. For women survivors, maternal healthcare offered by dispensary/hospital staff, in a serious time of need, constituted an important form of community help to them as did care by the matwon, a Traditional Birth Attendant.

Interactions between TBAs and dispensary/hospital staff were brought up by some participants from the matwon focus group as they related to receiving adequate help and care at a critical juncture. Matwon participants described feeling unwelcomed, humiliated and dismissed by healthcare staff upon arrival with a woman in a critical state. These TBAs perceived lack of respect for them and staff disregard for the urgency of the woman’s situation. They viewed these as contributing to the third kind of delay.

### Another delay: Of community to take responsibility

A community’s delay to take responsibility/be accountable for maternal death can also contribute to maternal mortality. For example, the issue of abortion and its complications were discussed by both groups of Community Leaders as they related to community responsibility. Participants spoke passionately about experiences of young women in the community with unwanted pregnancies. They also described women’s choices for induced abortion (illegal in Haiti) and related these to a partner’s insistence to abort a pregnancy because of a woman’s precarious socioeconomic status. They recounted devastating outcomes of hemorrhage and death when common illicit means were used to induce bleeding and terminate pregnancies. These means included self-medication of street drugs or ingestion of medicinal concoctions sold by the local boko (Voodoo sorcerer) or houngan (Voodoo male priest). A female Community Leader (CL3) reflected on maternal mortality as it relates to unwanted pregnancy, abortion and infection. She explained:

*Sometimes someone gets pregnant but it’s not the right time…she doesn’t want to tell her parents; she talks to her friends; her friends give her advice to take medicine, medicine that is too strong for her; it forces the baby out and after the baby is born she develops an infection, she doesn’t go to the hospital, she dies, she dies from the infection.* (CL3)

The status of the woman within the community and the impact of maternal death also emerged from discussions related to the fourth delay. While this topic was not broached with women survivors of near-miss experiences, it was discussed across four different focus groups: some participants described the coarse effects on motherless children, i.e. children without a father’s involvement would become uncared children then orphans and grow up as delinquents. Other participants cited a reduced socioeconomic status when women die since their community depends on women’s economic contribution. Many participants’ reflections related to community well-being and spoke of profound sadness when women die in their community. One leader described this:

*It’s very sad. Women aren’t supposed to die but we see women in our zone, they are pregnant and when the baby dies inside them, they can’t deliver the dead baby; after the delivery the woman dies herself; she’s not supposed to die, and it’s not good that women die because mothers give life and when they give life they aren’t supposed to lose their own lives- this is a sad time.* -male Community Leader (CL7)

In addition many focus group and interview participants discussed community social support with respect to the fourth delay. Some interview participants discussed the negative impact that social support might have on maternal outcomes. During discussions participants used examples of social support in which certain cultural/spiritual/religious beliefs had unintended negative impacts on women: A near-miss survivor described cultural beliefs and their effects when she sought the help of a boko leaf doctor in an attempt to relieve abdominal pain. She explained that she found no real help in the healer’s leaves, used up valuable resources and delayed getting medical healthcare, thereby negatively impacting her pregnancy outcome. This survivor participant recommended that problems of pregnancy were better left to treatment by a medical doctor. In another example, a woman’s partner spent precious money for the advice of a boko that delayed the woman reaching medical help, or obtaining it at all, or left her unable to pay for transportation to obtain essential healthcare services.

Participants also related examples of social support from partners, family members and neighbours which positively impacted maternal well-being. They described practical support as offered food, money or transportation for women. Women survivors appreciated supportive partners during labour or critical decision-making; neighbours who provided transportation; and family/friends who advised them to seek help/treatment (from matwon, traditional healer, healthcare provider). Community Leader participants included TBAs’ positive contribution to community responsibility vis-à-vis the fourth delay. They viewed these matwon as a vital link for women, as critical players who connect their community with healthcare providers and whose potential impact on maternal mortality in this community deserves close attention. Women survivors echoed this sentiment of positive support and added appreciation of their matwon who offered them food, tender care, trusted advice and physical/emotional support, and even accompaniment to hospital.

Finally, focus group and interview participants offered solutions to reduce maternal mortality in their community, and ways to reduce the fourth delay. These community solutions centered on prevention, community infrastructure and community mobilization. Participants suggested that prevention strategies should include more community education (for women and men, for teenagers/youth) related to Family Planning and birth spacing: consideration of a woman’s past pregnancy outcome(s), her current health, economic and social status, and cover topics such as pregnancy/birth/postpartum care, pregnancy danger signs, and the importance of receiving complete maternal healthcare. The need for more training of TBAs was suggested as another prevention strategy. Other solutions centered on improved community infrastructure including improved transportation infrastructure such as better roads and safer ways to transport women to hospital, and greater access to healthcare in their community which translated to more resources for TBAs/dispensary staff, more skilled dispensary staff and extended access to emergency care at the dispensary level. Participants also suggested community mobilization by way of the formation of Neighbourhood or Community Committees to directly address the problem of maternal mortality, and a coordinated communication relay/transport system (i.e. geographically, strategically-placed community stretchers, headlamps for carriers of women on stretchers fitted with security belts) in times of maternal health crisis.

Participants who survived near-miss maternal experiences offered solutions of a nature specific to the grave maternal circumstances they lived to tell. Their solutions included suggestions to seek appropriate care (i.e. care provided by a medical doctor and the avoidance of care/remedies by the boko or houngan), to seek care without delay (by calling on social support when needed, with the onset of danger signs and “earlier rather than later”), to choose an appropriate place of birth (recognizing that previous birth experiences play an important role in choice of birthplace) and to provide community support to pregnant, labouring and postnatal women (by the provision/sharing of food/financial support and participation of community members when needed for transportation, communication relay etc.).

### Being carried: A special example of the fourth delay

Many study participants from focus groups and interviews discussed women being transported on a board/door from their community in times of a maternal health crisis. The challenges of being carried were numerous and not simply a consequence of delays related to an individual’s decision to seek help, time to reach help, or provision of adequate care. “Being carried” constituted a particular example of the community’s involvement –and of their challenges created by the community’s own delay of responsibility for maternal death as they related to political will, mobilization and accountability.

Participants described the physical and logistical difficulties associated with getting a woman to the hospital, several hours walk away. Transportation of a woman (on a door) often involved locating four willing carriers (men), walking amidst challenging conditions and were also impacted by a family’s and/or matwon’s decisions to seek help from community members in the first place, and the time to reach help given physical and logistical transportation issues which had not been overcome. The scene is described in one male participant’s words:

*Carrying someone on our heads, [the path] isn’t wide enough for two people in front of each other to carry the lamp in the bucket so we need to walk one by one so we can see where we are going. Imagine if it is a pregnant woman who is in pain with labour and we drop her; they will both die, you see all of this problem why women die and they shouldn’t die because they are supposed to give life. We shouldn’t die under conditions like that.* –male Community Leader (CL7)

The timing of when to transport a woman from the community and the terrain that needed to be covered were impacted by community infrastructure. Focus group participants from each focus group and survivors of near-misses alike gave examples to illustrate the impact on maternal outcomes because of poor community infrastructure and lack of community engagement to overcome these obstacles. They suggested that as a community they are isolated because they are in a mountainous, hard-to-reach zone, and their dwellings are mostly distant one from the other.

*We don’t have roads, we don’t have roads. If we had roads, when someone has a problem, we could load them [on a vehicle], go to the hospital and then they don’t have time to die. But because we don’t have roads that means we have to wait until the morning to carry the person on our heads to the hospital…*-female Community Leader (CL6)

One woman survivor associated maternal death to several different delays and summarized it this way:

*Delays cause death; when you are pregnant you have the right [can] go to the doctor, and with every delay you can get sick; eclampsia can happen; but you might not find anyone to take you to the doctor’s; if you don’t have good roads they need to bring you on their heads to go to the hospital; delays can cause you to die because the roads aren’t made and there aren’t vehicles; if you have a [motorcycle] taxi number you can call it to come get you but the delay can cause you to die because you don’t have a road and so all you can do is go down the mountain on the carriers’ heads [on a door] and you can fall from the mountain when the carriers knock their feet on rocks and you slip with them [off the door*]. –Mother of Two Children (one stillborn and one neonatal death) (W2)

The community’s recognition that there has been a delay in their collective engagement to work together to identify ways within the community to improve their ability to transport women in crisis, mobilize resources already available and seek support to reduce poor maternal outcomes is clearly different from the other types of delays.

## Discussion

Maternal health and well-being in Haiti requires renewed actions to accelerate a reduction of reproductive health inequalities compared to other Latin American and Caribbean countries [5]. Haiti’s push toward safe motherhood continues to have its challenges related to unintended and adolescent pregnancies and abortion [19], provision of maternal health service among rural Haitian women, accessibility to antenatal care and skilled birth attendance and structural inequalities as they relate to socioeconomic determinants of maternal health (e.g. education, parity, distance from services) [12, 20]. Our study focused first on understanding community perspectives as to why women die during pregnancy, childbirth and the days after giving birth, and second on community-driven solutions to reduce maternal mortality in rural Haiti.

The delay in the decision to seek help (first delay) continues to be significant when maternal mortality is considered in rural Haiti. In agreement with prior research [12, 20] participants felt that women’s (especially youth) lack of knowledge/awareness, and misinformation about pregnancy/birth/afterbirth impacted maternal death in their community; they also identified the impact of economic status and how the status of the woman plays into maternal outcomes and women’s health-seeking behaviours [21]. Our participants connected these to an important community-identified concept of “negligence” and tied them to the first delay. Furthermore, our research overlaps with Weeks and team’s work where factors related to socioeconomic determinants of maternal mortality in Uganda were status of the woman, poor health education, limited resources and poor quality community health care [22].

Reaching help (second delay) during a maternal crisis also figured significantly in this rural community. Participants emphasized the need to improve community infrastructure- better roads, safer ways to transport women to hospital, closer access to care. These results support other literature regarding the second delay [11, 12, 22].

With respect to the third delay (to receive adequate care), we found the quality of interactions between the community and its healthcare resources and provision of them, impacted women in terms of receiving adequate help and care, much in alignment with other literature [22, 23]. While women survivors’ described positive interactions with matwon and/or dispensary/hospital staff, focus group participants described inadequate/incomplete/inappropriate healthcare services in their community, and viewed negative interactions with dispensary/hospital staff as contributing to a delay of the third kind. There is some overlap between our findings and research done in Ethiopia where community women’s and their partners’ perspectives were explored regarding decisions to deliver at home versus in health facilities [23]. Emergent themes from their work regarded client-related factors such as dependence on TBAs, and health system factors like poor welcome by staff [23] that contributed to the delay of women receiving adequate care.

Unique to our study, participants offered solutions to the first, second and third delays. Focus groups suggested the importance of community education, complete maternal healthcare, and Family Planning, a key form of maternal death prevention among teenagers/youth, and for other women attempting to use family planning and birth spacing for a variety of reasons. Women survivors of near-misses framed prevention by way of appropriate care, care sought without delay, appropriate choice of birthplace and provision of community support to pregnant, labouring and postnatal women. This aligns well with research on health-seeking behaviours and use of healthcare facilities [24]. Participants highlighted solutions and recommended optimal use of healthcare expertise and resources at the dispensary level and suggested the need for training of TBAs- committed accoucheurs already an integral part of the community whose expertise should be optimized to reduce maternal mortality.

Finally, in our study intimately related issues such as the status of the woman, women’s socioeconomic status and the social support of women emerged from discussions relating to maternal death. Participants referred to the community’s delay (and not just an individual’s delays) to take responsibility for reducing the number of women dying during pregnancy, childbirth and in the 42 days after birth. This seemed to be different from the other three delays and we believe it constitutes a fourth delay. The literature identifies two different types of fourth delay. One type is a delay related to the economic, psychological and physiological repercussions of near-miss maternal events; these repercussions are a consequence of a woman surviving a severe obstetric complication, and result in further maternal health complications and ongoing consequences for the woman and her family [9]. The second type corresponds to a community-based accountability for maternal (and newborn) health, and recognizes that the reduction of maternal mortality “takes a village” in order to affect change [10, 25]. This last type of fourth delay matches our findings most closely. No literature about maternal health in Haiti was located where specific reference was made regarding this notion of a fourth delay. However, it appears that many of this rural community’s challenges and participants’ solutions address a fourth delay- one that has the potential to impact individuals’ first, second and third delays. Together these emphasize the relevance of the community-based accountability type of fourth delay.

In terms of community accountability, focus group participants provided concrete examples of community mobilization as a strategic solution to reduce maternal mortality. In addition, the difficult experiences and practical solutions shared by women survivors of near-miss maternal experiences are valuable and insightful. This kind of proxy group of women, as described by Pacagnella and colleagues, represents a novel maternal group that often shares common characteristics as those who died “on the road of death” [9]. In our study, these novel participants framed community support through provision of food, money, and transportation from community members. Other researchers have also investigated near-misses in different low-middle income settings, and their impact on subsequent pregnancies [26]. They have considered the perspectives of women survivors in terms of women’s bodily integrity, household economy and social identity/stability as a result of their near-loss of life experiences [27], and women’s fear of dying, loss of their baby and how the quality of care they received could impact future health-seeking behaviour/use of healthcare facilities [24]. In our study the valuable contribution of these women’s unique perspectives has provided a better understanding of the socioecological determinants of maternal death and solutions needed to reduce maternal mortality in this rural Haitian community of which most solutions point to the fourth delay.

In terms of maternal wellness, we found this fourth delay to affect the first, second and third delays, and to be impacted by the community’s knowledge, political will, accountability, mobilization and empowerment. Through local ownership and engagement in our study we found that community knowledge and awareness of maternal death in the community impacted the political will to mobilize and find solutions to reduce maternal mortality and in turn created a sense of empowerment whereby positive change could be realized.

The CBAR design of our research strengthened this study by way of encouraging community learning, building project integrity and accountability, and bringing about community mobilization. This capacity-building allowed for better understanding of maternal mortality in this rural Haitian community that may provide valuable insight for future interventions that are community-based, community-driven and sustainable.

The study was limited by small sample size and selective inclusion of key community members’ perspectives regarding maternal mortality. Consequently, findings from this research are not representative and should not be generalized to all rural (Haitian) communities. Future explorations should include the missed perspectives of under-represented community members such as teenagers/youth, traditional healers, elders of the community, religious leaders, dispensary/hospital staff and administration, and policy makers. The authors must also consider a possible bias in drawing conclusions with respect to the fourth delay as part of our conceptual framework.

## Conclusion

This study highlights the indigenous knowledge and experience of members of a rural Haitian community as they relate to maternal mortality in their community. Community perspectives on maternal death are important in Haiti. A deeper exploration regarding the important community-identified concept of negligence is needed in order to better understand the implications of individual and community accountability for maternal health and well-being, and community health at large, and how these overlap the first and fourth delays and potentially impact the other delays as well. Finding sustainable ways to reduce maternal mortality requires a community-based/centred and community-driven comprehensive approach to maternal health/well-being. This includes the engagement of community members that is dependent upon community knowledge, political will, mobilization, accountability and empowerment. An engaged and empowered community is one that is well placed to find ways that work in their community to reduce the fourth delay and in turn maternal death. Potentially, community ownership of the challenges and the solutions can lead to more sustainable improvements in maternal health/well-being in Haiti.

## Abbreviations

CBAR: Community-Based Action Research
CL: Community Leader
CRT: Community Research Team
FGD: Focus Group Discussion
Htn: Haitian
II: Individual Interview
TBA: Traditional Birth Attendant
W: Woman (survivor of near-miss maternal experience)
